# Impact of amino acid supplementation on hydroponic lettuce (*Lactuca sativa* L.) growth and nutrient content

**DOI:** 10.1038/s41598-025-00294-x

**Published:** 2025-05-06

**Authors:** Shumaila Khan, Muhammad Zafar Iqbal, Farheen Solangi, Shahid Azeem, Muhammad Adnan Bodlah, Muhammad Saqlain Zaheer, Yasir Niaz, Muhammad Ashraf, Muhammad Abid, Hera Gul, Hongjun Yu, Qiang Li, Jiang Weijie, Muhammad Rizwan, Salim Manoharadas

**Affiliations:** 1 Institute of Vegetables and Flowers, Chinese Academy of Agricultural Sciences (CAAS), No.12 Zhongguancun South Street, Haidian District, Beijing, 100081 China; 2 Climate Smart Agriculture Accelerator Program (CSAAP) KFUEIT GIZ SAR, Rahim Yar Khan, Pakistan; 3 Department of Agricultural Engineering, Khwaja Fareed University of Engineering and Information Technology, Rahim Yar Khan, Pakistan; 4 State Key Laboratory for Conservation and Utilization of Bioresources in Yunnan, Yunnan Agricultural University, Kunming, 650201 China; 5 Research Centre of Fluid Machinery Engineering and Technology, Jiangsu University, Zhenjiang, 212013 Jiangsu China; 6 Adaptation to Climate Change, Deutsche Gesellschaft für Internationale Zusammenarbeit (GIZ) GmbH, Bonn, Germany; 7 Department of Horticultural Sciences, Faculty of Agriculture & Environment, The Islamia University of Bahawalpur, Bahawalpur, 63100 Pakistan; 8 Department of Plant Nutrition, Institute of Crop Science and Resource Conservation (INRES), University of Bonn, 53115 Bonn, Germany; 9 Department of Botany and Microbiology, College of Science, King Saud University, 11451 Riyadh, Saudi Arabia

**Keywords:** Amino acids, Hydroponic lettuce, Lactuca sativa L., Plant growth, Nutrient content, Amino acid supplementation, Plant sciences, Agroecology

## Abstract

Lettuce (*Lactuca sativa* L.), a widely cultivated leafy green, is valued for its rich content of bioactive compounds, including folates, vitamins, tocopherols, ascorbic acid, and antioxidants. This study aimed to evaluate the effects of amino acid supplementation on the growth and nutrient content of hydroponically grown lettuce. A greenhouse experiment using a completely randomized design (CRD) was conducted, with three replications and three plants per replication. There were 4 treatments (T_0_ (Control), T_1_ (Methionine 20 mg/L), T_2_ (Tryptophan 220 mg/L, T_3_ (Glycine 200 mg/L) of this experiment Growth parameters, including biomass, leaf length, leaf width, and leaf area, were measured four weeks after transplantation. L-methionine supplementation resulted in a significant improvement in plant growth, with a 23.60% increase in biomass and a 31.41% increase in leaf area. Conversely, L-tryptophan treatment led to substantial reductions in growth, including a 98.78% decrease in biomass. Nutrient analysis revealed that amino acid treatments, especially methionine, enhanced the nitrogen, phosphorus, and potassium content in leaf tissues. These results suggest that L-methionine has a positive effect on both growth and nutrient uptake in hydroponic lettuce, while L-tryptophan and L-glycine negatively affect plant development. The differential responses to amino acid treatments may be attributed to their distinct roles in plant metabolism, with methionine enhancing sulfur-containing compounds and proteins essential for growth, while tryptophan and glycine could disrupt metabolic pathways. Future research should explore the mechanisms underlying these effects and evaluate the optimal amino acid concentrations for maximizing hydroponic lettuce production and nutrient density.

## Introduction

Lettuce is ranked among the most well-known leafy vegetables crops due to its demand in the world market^1^. In regard to lettuce consumption, China is occupying a significant position as the world’s largest producer. However, the regular inclusion and consumption of raw lettuce and other green vegetables in coming up spicy soups and other recipes of Chinese cuisine can pose great health hazards and some of the most worrisome aspects entail pesticide residues^2^. Pesticide-free products are considered ideal and organic foods especially those good for consumptions in fresh salads have been promoted in the market. There has been the rising demand in the production of vegetables with improved health-promoting qualities and at the same time requiring less input to be produced^1,3^. This strategy plans to increase the nutritional value of the crops produced without reducing the production rate or having adverse effects on the growers. Hints for further investigation might be sought by understanding how plants act based on nitrogen availability, thus supporting the sustainable development in the management of the crop plants^3^.

Nitrogen (N) is one of the most important minerals required for plant growth and is integral for the development, expansion, growth, and reproduction of plants. It makes up approximately three to four% of the above ground plant biomass. It is a fundamental building block of both structure and function in plants. Nitrogen is a key element in the structural and functional composition of plants, and plants cannot survive without it^1,3^. The major N source in soil is nitrate (NO_3_^–^). Plant growth is most frequently limited by the availability of NO_3_^– 4^.

The growth of lettuce reportedly increases when the external N supply increases. Although 78% of our atmospheric nitrogen is available in the form of gas, plants cannot absorb it directly from the air but can absorb it only in specific usable forms, which are naturally limited in soils^5^. Therefore, plants are unable to flourish to their maximum potential without the application of supplemental nitrogen fertilizer^6,7^. In general, fertilizers are necessary because natural soils cannot always provide the amount of nutrients that are required for optimal plant growth^8^. According to recent studies, organic fertilizers can be a viable and practical substitute for mineral fertilizers when lettuce is grown in fields^8,9^. Therefore, amino acids are very simple and soluble in water and are a small source of nitrogen; however, very little information about the use of amino acids as an additional source of organic nitrogen is available^1,8^.

One study reported that applying a glycine (amino acid) solution to lettuce plants and soil was as efficient as using inorganic nitrogen fertilizers, but it did not clarify the distinct effects of foliar versus soil application^10^. The use of mixed amino acid solutions on lettuce grown in fields has not been the subject of any notable study. Several crops have been shown to grow and produce more vigorously when tryptophan (an amino acid) is applied exogenously as a precursor of auxin^10,11^. When added to soil, tryptophan can increase auxin production by soil microbes because of its role as an auxin precursor. Compared with pure auxin, L-TRP has been shown to have superior effects on seed germination, nutrient absorption, growth and yield in higher plants^11^.

Plants that obtain fertilization with amino acids might conserve a significant amount of energy, which may have been essential for the synthesis of amino acids and for food absorption; these plants bypass the need to synthesize large molecules from smaller ones, conserving energy that would otherwise be used^12,13^. This saved form of energy can be helpful in nutrient transportation, increasing the number of energy molecules, increasing yields, increasing insect and pest resistance, and improving the quality of production, thus reducing overall plant stress^12,14,15^. Owing to their chelating ability and solubility in water, amino acids can be considered an efficient form of nitrogen fertilizer for plants. Since the chains of amino acids do not need to be further broken down or shipped out, they can therefore be utilized promptly as a fundamental component for growth and development^14^.

Fertilization is believed to be essential for the successful production of lettuce crops^11^. Amino acids may be taken up directly by the roots, and they also have a beneficial interaction with micronutrients in the rhizosphere that helps the roots absorb them^14^. Global food output has improved dramatically in recent years because of the adoption of hydroponics, which offers better control over climatic and insect variables, as well as more effective use of water and nutrients^1^. Hydroponic farming also yields higher-quality and more productive crops, which boosts economic revenues and competitiveness^16^. Despite the growing interest in hydroponic cultivation systems and the potential benefits of amino acid supplementation, there is limited research on their specific effects on lettuce growth and nutrient content. Amino acids, as vital components of plant metabolism, offer an alternative source of nitrogen, which is essential for optimal plant development. Previous studies have demonstrated the positive effects of amino acids on various crops; however, their impact on hydroponically grown lettuce remains poorly understood. This research aims to address this gap by evaluating the effects of three amino acids—L-tryptophan, L-glycine, and L-methionine—on the growth and nutrient content of hydroponic lettuce. By examining the different responses to these amino acids, this study seeks to contribute to optimizing hydroponic lettuce production and provide insights into improving both growth and nutritional quality in a controlled environment.

## Materials and methods

This research was carried out at the Graduate School of the Chinese Academy of Agricultural Sciences (GSCAAS) glasshouse in Haidian district in Beijing, China. The experiment was conducted in a greenhouse to determine the effects of three amino acids at the following levels (Table 1). Lettuce seeds were acquired and purchased from the “Seed Technology Institute of Vegetables and Flowers, CAAS, Beijing, China. The lettuce seeds were sown in germination trays filled with a peat moss-based growing mixture, at a density of 2–3 seeds per hole. The greenhouse environment was maintained at an average monthly temperature of 24°C during the night and 34°C during the day throughout the experimental period. Following seed emergence, the seedlings were watered daily with tap water for the first 10 days. From day 10 onward, the plants received weekly irrigations with a Hoagland’s nutrient solution. At approximately 30 days after sowing (DAS), the seedlings were transferred to a hydroponic system designed for water-cycled cultivation. No amino acid treatments were applied during the germination or seedling stages. The amino acids were introduced only after transplanting, directly into the circulating hydroponic nutrient solution, beginning eight days after transplantation. To acclimate the plants to the new environment, at least two fully expanded leaves were submerged in the hydroponic medium, ensuring their proper establishment in the system. The greenhouse temperature was controlled to maintain an average of 24°C during the day and 34°C at night. Humidity levels ranged between 50–60%, and the light cycle was set to 12 hours of light and 12 hours of darkness daily.

**Table 1:** Concentrations of diverse amino acids applied to lettuce plants as roots in hydroponic solution.

Experiment No.	Amino acid	Concentration (mg/L)
T_0_	Control	Control (0.0)
T_1_	Methionine	20
T_2_	Tryptophan	220
T_3_	Glycine	200

### Plant material and growth conditions

The plantlets were grown in solution supplemented with 75% strength Hoagland’s nutrient mixture (Table 2) for lettuce as described previously^1^. The water tanks, which had a capacity of 80 L per gutter, were checked and adjusted to pH 5.8–6.3 and EC 1.5–2.0 mS^− 1^ on a daily basis to maintain optimal growth conditions, as lettuce has very delicate leafy plants. Although the EC for the 3/4X Hoagland’s solution is typically 1.5 dS/m, the EC was adjusted to 2 dS/m to ensure adequate nutrient availability, particularly for amino acid supplementation. The culture mixture was replaced with fresh medium at seven-day intervals during the crop season. To prevent nutritional shock, amino acid supplementation was initiated eight days after transplantation, by adding the amino acids directly into the hydroponic nutrient solution. The peat moss used for germination trays had a pH of 6.2 and a particle size distribution of 0.1–1 mm, providing optimal water retention and aeration for lettuce seeds. The trays were placed under controlled conditions with a light intensity of 200 µmol m⁻²s⁻¹. The concentrations of amino acids were 20 mg/L for methionine (T_1_), 220 mg/L for tryptophan (T_2_), and 200 mg/L for glycine (T_3_). The hydroponic system employed was a nutrient film technique (NFT) with water circulation maintained by an air pump providing adequate oxygenation.

### Vegetative growth characteristics

During growth, the following biometrical parameters of the plants were measured every week beginning seven days after treatment (DAT). The procedures proposed by previous researchers^17,18^ were employed in the data processing at each time for the quantity and quality of the leaf height and diameter of the lettuce plants with the leaf area with the following formula.

$$\:AF\:\left(cm2\right)\:=\:0.7\:\times\:\:Length\:\left(cm\right)\:\times\:\:Width\:\left(cm\right)\:-2.4$$

A measuring scale was used to record the leaf quality parameters, such as length and width.

To measure the roots of the plants, the plants were removed from the nutrient mixture, the roots were detached, placed and blotted on paper, the length was measured in centimeters with a measuring tape, and the length of the roots was calculated. Prior to the study analysis, an electronic balance (S = 0.1 g) ACCULAB V-1200 was used to standardize the error distribution as stated previously^19^. The roots of the gathered plants were thoroughly cleaned with distilled water, blotted onto filter paper, and then dried in an oven at −45 °C until they were completely dry for dry weight^17,20^.

The relative growth rate (RGR) index was computed via the following formula:

$$\:RGR\:=\:(lnW2\:\--\:lnW1)(t2\:\--\:t1),$$

Where W2 and W1 represent the plant dry mass (g) at periods t2 and t1, respectively.

The net assimilation rate was computed via the following formula:

$$NAR~ = ~dW/\left( {A \cdot dt} \right),$$

Where dW is the dry mass increment (g), dt is the culture duration (days), and A is the area of assimilation organs (dm^2^.

**Table 2:** Lettuce culture nutrition solution formula for hydroponics (mg/L).

Chemical name	Concentration (mg/L)
Calcium Nitrate (Ca(NO_3_)_2_)	1122
Potassium nitrate (KNO_3_)	910
Potassium dihydrogen phosphate (KH_2_PO_4_)	272
Ammonium nitrate (NH_4_NO_3_)	40
Magnesium sulphate (MgSO_4_·7 H_2_O)	247
Iron chelate(Fe-EDTA)	16.80
Zinc sulfate (ZnSO_4_·7 H_2_O)	1.20
Boric acid (H_3_BO_3_)	0.28
Copper sulphate (CuSO_4_·5 H_2_O)	0.20
Sodium molybdate(Na_2_MoO_4_·2 H_2_O)	0.10
Manganese sulphate (MnSO_4_·H_2_O)	0.86

### Photosynthesis measurements

The total chlorophyll content was measured via a portable SPAD apparatus (SPAD-502 plus by Konica Minolta, INC. Japan). Chlorophyll fluorescence measurements were used to assess the transpiration rate of the plants in terms of their photosynthetic efficiency directly on the leaves of each plant via a gas exchange system with three leaves per replication^21^.

### Nutrient contents

Using a gas exchange device, measurements of chlorophyll fluorescence were used to evaluate each plant’s rate of transpiration and photosynthetic efficiency directly on its leaves. The leaf samples were dried, pulverized, and digested in HNO_3_ using a high-pressure digestion system (Thermo Scientific). Nitrogen content was determined using the Kjeldahl method, and the total nitrogen content was measured using an Optima 5300 DV optical emission spectrometer with standard calibration procedures^22^.

### Statistical analysis

In line with the experimental design, the recorded data of three replicates for the parameters under study under various treatments were subjected to analysis of variance (ANOVA) via the IBM SPSS statistics 20 software package to quantify and analyze the causes of variation. Superscripts indicate the ranks in the pertinent tables. (DMRT) Duncan’s multiple range test (*p* = 0.05) was used to evaluate the mean performances of various treatments for the particular parameters under consideration.

## Results

**Fig. 1:**
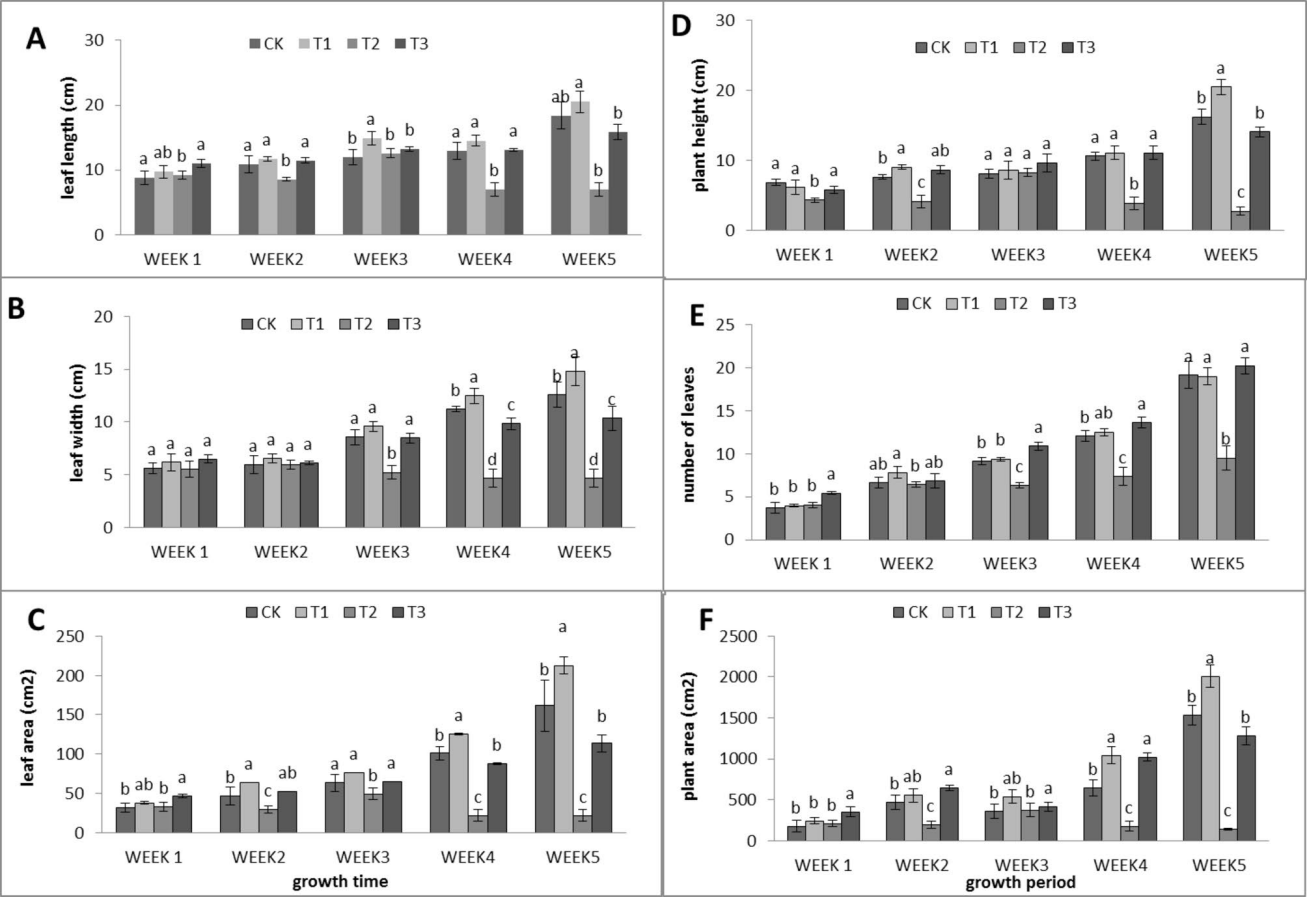
Leaf length (**A**), leaf width (**B**), leaf area (**C**), plant height (**D**), number of leaves (**E**), and plant area (**F**). According to the Duncan multiple range test at the 5% significance level, means with identical lowercase letters are not significantly different.

The effects of the three amino acid treatments on lettuce growth exhibited distinct patterns. Methionine treatment generally led to positive effects on plant growth, including increased leaf size, root length, and overall plant fresh and dry weights. In contrast, glycine and tryptophan treatments were associated with reduced growth parameters, with tryptophan particularly showing negative impacts on leaf size, plant height, and fresh weight. These trends were consistent across various growth stages and measurements. In the 1st week, plants treated with nutrient mixture modified with amino acids (Hoagland + amino acids) presented diverse responses as time progressed toward the maturation of the plants. Compared with those of the control plants, the length of the leaves of the methionine-treated plants increased by 11.41%, whereas the lengths of the leaves of the glycine- and tryptophan-treated plants decreased by 13.76% and 61.92%, respectively (Fig. 1A). Furthermore, leaf width increased by 17.46% following treatment with methionine but decreased by 18.25% and 63.49% following treatment with both glycine and tryptophan, respectively (as shown in Fig. 1B).

The leaf area increased significantly in response to methionine by 31.41%, whereas glycine and tryptophan decreased the leaf area by 29.67% and 86.25%, respectively (Fig. 1C). Compared with the control, tryptophan decreased plant height by 82.91% (Fig. 1D). Compared with the control, tryptophan significantly decreased the leaf number by 50.36% (Fig. 1E). Compared with the control treatment, methionine had an encouraging effect on the plant area of 30.84%, but the contents of glycine and tryptophan both decreased by 16.49% and 90.78%, respectively, when no amino acids were present (Fig. 1F).

**Fig. 2:**
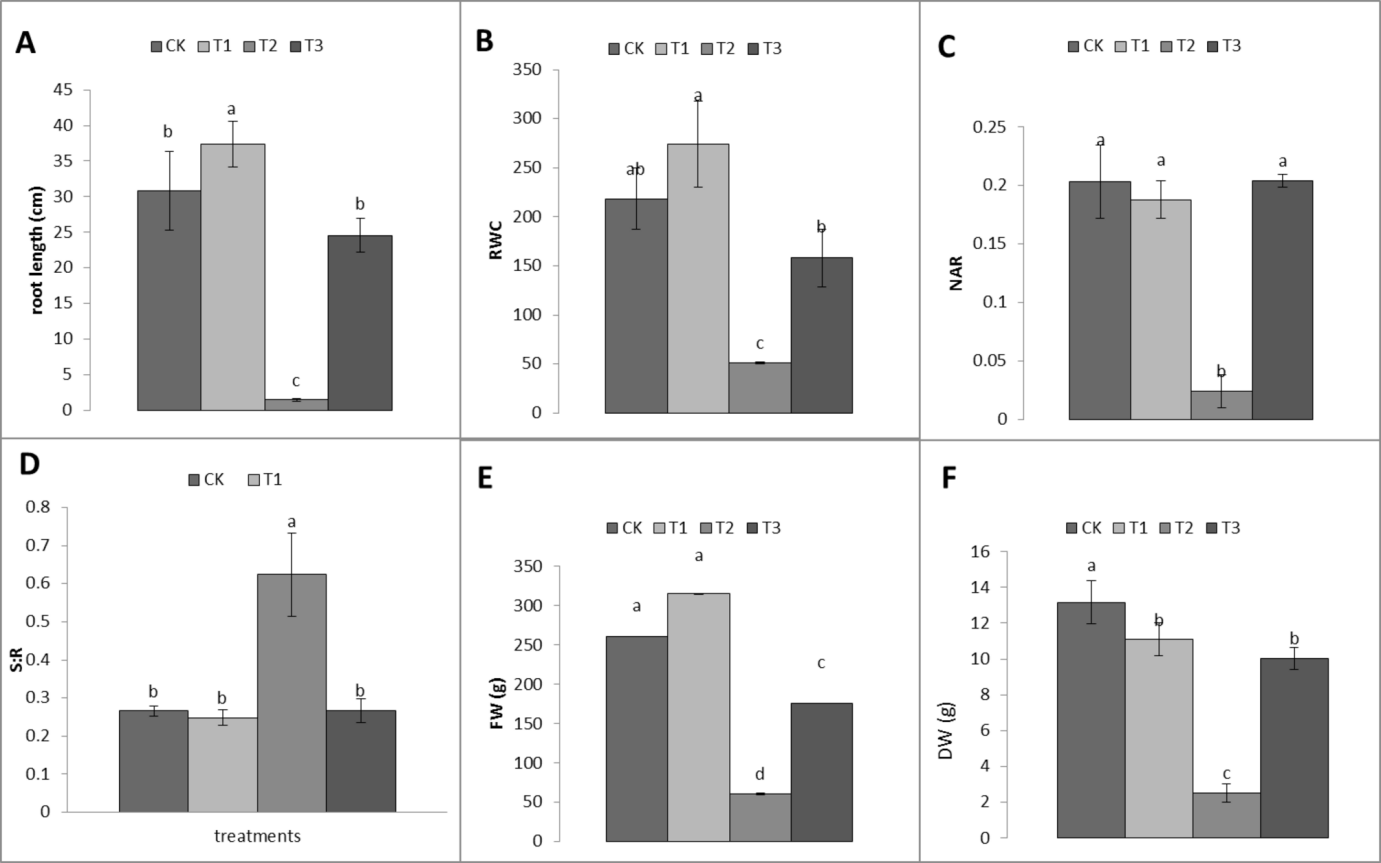
Analysis of how different amino acids affect the length of roots (**A**), (S: R) shoot–root ratio (D), (RWC) relative to the water content (**B**), and (NAR) net assimilation rate (**C**), root-to-shoot ratio (**D**), Plant fresh weight (FW) (**E**), dry plant weight (DW) (**F**).

Figure 2 shows that root length significantly increased (*P* ≤ 0.05) with methionine application but decreased when plants were treated with tryptophan (Fig. 2A). The relative water content was strongly affected by tryptophan treatment but increased (*P* ≤ 0.05) by methionine (Fig. 2B). The net assimilation rate was negatively correlated with tryptophan application (Fig. 2C), whereas the root-to-shoot ratio was significantly (*P* ≤ 0.05) high for tryptophan (Fig. 2D). The plant fresh weight significantly (*P* ≤ 0.05) increased by 20.88%, whereas it decreased by 32.50% and 76.74% with the application of glycine and tryptophan, respectively (Fig. 2E). The dry weight of the plants significantly (*P* ≤ 0.05) increased by 15.71% when methionine was added and decreased by 80.86% when tryptophan was applied (Fig. 2F). The data in Table 3 show that while all amino acid treatments increased chlorophyll levels, tryptophan application substantially (*P* < 0.05) lowered the photosynthesis rate and transpiration rate. Table 4 shows that after tryptophan treatment, the leaf area indices SLA and LAR and the leaf dry matter contents were highly significantly different (*P* < 0.05). There was no significant difference in the relative growth rate of the plants in the methionine and tryptophan groups, but the relative growth rate of the glycine group was markedly different from that of the control group. When the ascorbic acid readings were compared with those of the control, amino acids had no effect. The DM percentage was significantly (*p* ≤ 0.05) high when the plants were treated with tryptophan (Table 5).

**Table 3:** Effects of different amino acids on the net photosynthesis rate (Pn) and total transpiration rate (Tr) of plants.

Treatments	Pn	Tr	SPAD
Control	7.4a	1.83a	16.5a
Methionine (Meth)	5.1ab	1.03ab	16.7a
Tryptophan (Try)	2.3c	0.70b	15.6ab
Glycine (Gly)	4.6b	1.54a	17.4a

According to Duncan’s multiple range test at the 5% significance level, means with identical lowercase letters show no significant variations.

**Table 4:** Effects of various amino acids on the specific leaf area (SLA), leaf area ratio (LAR), leaf dry matter content (LDMC), root mass ratio (RMR), and relative growth rate (RGR) of plants.

Treatments (mg/L)	LAI (dm^2^ dm^− 2^)	LDMC	RMR	SLA (dm^2^ g^− 1^)	LAR	RGR (g.g^− 1^.d^− 1^)
CK	2.73b	0.03b	0.01b	0.06ab	22.67ab	4.44b
Methionine	2.06b	0.05b	0.01b	0.04ab	6.38c	1.18c
Tryptophan	26.18a	0.58a	0.15a	0.10a	63.65a	1.34c
Glycine	3.85b	0.05b	0.01b	0.07ab	35.78b	7.31a

According to Duncan’s multiple range test at the 5% significance level, means with identical lowercase letters show no significant variations.

Table 5 presents the influence of various amino acids on the proportions of ascorbic acid and dry matter in lettuce leaves. The ascorbic acid content did not differ significantly across treatments, with control, methionine, and glycine showing similar levels (0.25 to 0.3 mg/g), while tryptophan had a slightly lower value of 0.2 mg/g. In terms of dry matter content, significant differences were observed. Tryptophan-treated plants exhibited the highest dry matter content at 32.12%, followed by glycine with 24.7%, while methionine treatment resulted in a lower dry matter content of 10.1%. The control plants had a dry matter content of 13.4%. The data in Table 6 present the response of the nitrogen, phosphorus and potassium contents to the different amino acid treatments. The results indicate that treating plants with methionine and tryptophan significantly (*P* ≤ 0.05) increased the nitrogen content in the leaf tissues compared with the control plants. However, glycine did not result in a significant increase in nitrogen content, and it showed lower nitrogen levels compared to methionine and tryptophan (Table 6).

**Table 5:** Influence of various amino acids on the proportions of dry matter and ascorbic acid in leaves.

1	Treatments	Ascorbic acid	Dry matter (%)
2	Control	0.25a	13.4bc
3	Methionine	0.3a	10.1c
4	Tryptophan	0.2a	32.12a
5	Glycine	0.3a	24.7b

According to Duncan’s multiple range test at the 5% significance level, means with identical lowercase letters show no significant variations.

**Table 6:** Effects of various amino acids on the macro- and micronutrients in lettuce.

Treatments	*N*	*P*	K	S	Ca	Mg	Fe	Cu	Mo	Na	Zn	Al
Control	1.4b	12.3d	208.1b	9.8b	93.7a	22.9a	6.8a	0.10c	0.16b	18.25a	0.2c	13.1a
Methionine	4.3a	32.2b	420.7a	10.1b	66.3c	12.6b	2.1b	0.06c	0.07c	4.48b	0.4a	7.1b
Tryptophan	3.7a	36.8a	421.4a	13.1 a	81.5b	13.4b	2.8b	0.14b	0.1bc	3.95b	0.4a	9.0b
Glycine	4.3a	21.7c	230.8b	13.3a	44.6d	12.6b	6.9a	0.23a	0.20a	3.40b	0.3b	9.6ab

According to Duncan’s multiple range test at the 5% significance level, means with identical lowercase letters show no significant variations.

## Discussion

The application of amino acids as fertilizers represents a promising approach to enhancing crop growth and nutrient content, particularly in hydroponic systems^16^. Amino acids, as fundamental components of proteins, play a pivotal role in various physiological and metabolic processes essential for plant development. In this study, we investigated the effects of individually applied amino acids L-tryptophan, L-glycine, and L-methionine on the growth and nutrient profile of hydroponically grown lettuce (*Lactuca sativa* L.)^1,16^. Our results demonstrate that these amino acids influence growth patterns and nutrient uptake in distinct ways, with methionine showing the most favorable outcomes. These findings underline the potential of targeted amino acid supplementation to optimize nutrient assimilation and improve lettuce productivity, offering insights into sustainable agricultural practices^2^. Amino acids are essential for the development and growth of lettuce plants when used as fertilizers. They are the building blocks of proteins, which are crucial for various physiological functions in plants. When applied as fertilizer, amino acids significantly increase the uptake and assimilation of nutrients, leading to improved plant health and productivity^23^.

Nitrogen, a key component of amino acids, is vital for plant growth. Nitrogen is mostly absorbed by plants from the soil as nitrate (NO3-) and ammonium (NH4+), which are then converted into proteins and other important compounds within the plant. By providing amino acids directly, plants can more efficiently utilize nitrogen, resulting in faster growth and higher yields^24^. In addition to improving nitrogen efficiency, amino acids contribute to several metabolic processes, including hormone biosynthesis, defense mechanisms, and the formation of secondary metabolites^14^. They also aid in the synthesis of crucial molecules such as enzymes, vitamins, and purines, which support overall plant health. Furthermore, amino acids can increase photosynthesis and protect plants from stress, leading to more vigorous and resilient growth^25^.

The use of amino acids as fertilizers also has environmental benefits. They can reduce the need for synthetic fertilizers, which are often associated with environmental pollution due to nitrate leaching and runoff. By promoting the efficient use of nitrogen and other nutrients, amino acid-based fertilizers help minimize these negative impacts while maintaining high crop productivity^19^. Moreover, amino acids can form complexes with metal ions, increasing the bioavailability of essential minerals to plants^26^. This enhances nutrient uptake and further supports healthy growth. By increasing water and mineral absorption, the use of amino acids in fertilizers has been demonstrated to increase the growth and productivity of a variety of crops, including lettuce^27^.

Although the administration of amino acids to horticultural crops is a frequent technique globally, most research has focused on biostimulants containing a blend of amino acids^28^. In our study, only amino acids provided individually were used to regulate growth parameters associated with the metabolism of nitrogen in lettuce. On the basis of our results, we can suppose that the amount of N supplied by the root application of methionine increases the already present N in the leaf contents, which is approximately equal to 1% of the amino acid content^29^. Therefore, the use of methionine applied to roots is not a source of nitrogen for plants; instead, methionine behaves as a signaling molecule in diverse processes involved in metabolism, initiating more protruding absorption of nitrogen and sulfur by plants^30^.

Depending on their demand, varietal selection, developmental stage plants can utilize amino acids. This might be the cause of inconsistent responses among plants. There are various beneficial effects that can be described by methionine. First and foremost, this amino acid can play a crucial role in the protein preservation structure required for cell growth, cell division and differentiation^31^. Furthermore, methionine can make essential nitrogen and sulfur available to plants according to their demand, and finally, it has the capacity to transform into polyamines and extend into hormonal structures, allowing nitrogen to be dispersed into cells and organs^32^. Methionine also acts as a buffer and potential source to provide energy and carbon, precursors for the biogenesis of spermidine and gibberellin^33,34^. In addition to being necessary for the formation of hairy roots, methionine functions as a growth regulator by acting as a cytokinin, auxin, and brassinosteroid, which promotes the initiation of root formation and helps the plant absorb more nutrients^35,36^.

The increased chlorophyll contents might be due to the availability of a beneficial level of methionine, which is necessary for anabolic processes, and to enhancements in metabolic efficiency in the plant, as described earlier^37,38^, increasing cell formation, increasing fresh and dry matter and increasing different growth criteria^39^. It is important to consider the role of electrical conductivity (EC) in this hydroponic system, as fluctuations in EC can impact nutrient availability and uptake efficiency. While EC was maintained within optimal ranges throughout this experiment, subtle variations in EC levels may have influenced plant responses, particularly in the uptake of essential nutrients such as nitrogen and phosphorus. Future studies should investigate how EC fluctuations might interact with amino acid treatments to further optimize plant growth and nutrient absorption in hydroponic systems. This could provide valuable insights into improving the precision and efficiency of hydroponic cultivation, especially for crops like lettuce. The better growth and development of crops can be associated with the amount of precursor-released auxins and the crop type and variety^40^. The impact of tryptophan (an amino acid) on yield and growth could also be attributed to (a) the overproduction of auxin metabolites that are harmful to plants^27,31^. (b) Tryptophan caused excess auxin production, which was then absorbed by the plant roots. Alternatively, direct absorption by plant roots may prevent the roots from absorbing nutrients. (c) Changes caused by the addition of L-TRP to the rhizosphere microbial population might affect yield and growth^41^. The findings from this study highlight the significant potential of methionine as a growth-enhancing supplement in hydroponic lettuce cultivation, owing to its role in nitrogen metabolism, hormonal regulation, and stress mitigation. While L-tryptophan exhibited inhibitory effects likely linked to auxin overproduction or microbial interactions in the rhizosphere, methionine consistently enhanced growth and nutrient uptake^1,35^. These outcomes emphasize the importance of selecting appropriate amino acids based on plant species, developmental stage, and environmental conditions. By integrating amino acid-based fertilizers into hydroponic and conventional farming practices, we can promote sustainable agriculture while minimizing environmental impacts. Our findings align with previous research that has suggested amino acids, particularly methionine, can enhance growth and nutrient uptake in various crops. For instance, studies by Khan et al.^1^ demonstrated that methionine improves nitrogen assimilation and supports root growth in hydroponically grown lettuce, which is consistent with our observations. In this study, the correlation between the application of amino acids and growth parameters was clearly evident. For example, methionine, which enhanced growth parameters such as leaf area and plant height, was associated with improved nitrogen uptake and chlorophyll content, suggesting that methionine plays a critical role in nitrogen metabolism and photosynthesis efficiency. The increased dry matter percentage observed in tryptophan-treated plants (32.12%) was closely correlated with the enhanced leaf area and chlorophyll content, although this was not reflected in improved overall growth, indicating that while tryptophan may stimulate certain metabolic processes, it could also lead to the overproduction of auxin metabolites, which may inhibit further growth. Additionally, changes in nutrient contents, such as nitrogen and phosphorus, were significantly correlated with the amino acid treatments, particularly with methionine, which facilitated more efficient nitrogen assimilation and, in turn, supported better plant development. This relationship highlights the importance of considering the interaction between amino acids and essential nutrients when optimizing hydroponic systems for crop growth. Future studies should explore the molecular mechanisms behind these correlations to better understand how amino acid supplementation influences both growth parameters and nutrient profiles.

## Conclusions

This study demonstrates that the supplementation of specific amino acids can significantly influence the physical performance and growth parameters of hydroponically grown lettuce (*Lactuca sativa* L.), with responses varying depending on the type of amino acid applied. Methionine notably enhanced lettuce growth, increasing leaf width by 17.46% and plant area by 30.84% compared to the control. Conversely, tryptophan exhibited a negative effect, reducing leaf width and plant area by 63.49% and 90.78%, respectively. These findings highlight methionine as an effective growth stimulant for hydroponic lettuce cultivation, offering potential for improving crop productivity in controlled environments. Further investigations are recommended to explore the underlying mechanisms and optimize amino acid application strategies for diverse crop systems.

## Data Availability

The datasets generated and analyzed during the current study are not publicly available but can be obtained from the corresponding author (J.W.) upon reasonable request.
